# Identification of the Ovine Keratin-Associated Protein 26-1 Gene and Its Association with Variation in Wool Traits

**DOI:** 10.3390/genes8090225

**Published:** 2017-09-13

**Authors:** Shaobin Li, Huitong Zhou, Hua Gong, Fangfang Zhao, Jiang Hu, Yuzhu Luo, Jon G. H. Hickford

**Affiliations:** 1 Gansu Key Laboratory of Herbivorous Animal Biotechnology, Faculty of Animal Science and Technology, Gansu Agricultural University, Lanzhou 730070, China; lisb@gsau.edu.cn (S.L.); zhouh@lincoln.ac.nz (H.Z.); zhaofangfang@gsau.edu.cn (F.Z.); huj@gsau.edu.cn (J.H.); 2 International Wool Research Institute, Gansu Agricultural University, Lanzhou 730070, China; hua.gong@lincoln.ac.nz; 3 Gene-marker Laboratory, Faculty of Agricultural and Life Sciences, Lincoln University, Lincoln 7647, New Zealand

**Keywords:** keratin-associated protein KAP26-1 gene (*KRTAP26-1*), variation, wool traits, sheep

## Abstract

Keratin-associated proteins (KAPs) are structural components of wool and hair fibres, and are believed to play a role in defining the physico-mechanical properties of the wool fibre. In this study, the putative ovine homologue of the human KAP26-1 gene (*KRTAP26-1*) was sequenced and four variants (named *A–D*) were identified. The sequences shared some identity with each other and with other *KRTAPs*, but they had the greatest similarity with the human *KRTAP26-1* sequence. This suggests they represent different variants of ovine *KRTAP26-1*. The association of these *KRTAP26-1* variants with wool traits was investigated in the 383 Merino-Southdown cross sheep. The presence of *B* was associated (*p* < 0.05) with an increase in mean fibre diameter (MFD), mean fibre curvature, and prickle factor (PF). The presence of *C* was found to be associated (*p* < 0.05) with an increase in wool yield (Yield) and mean staple length (MSL), and a decrease in MFD, fibre diameter standard deviation (FDSD), and PF. The results suggest that sheep with *C* have, on average, higher wool quality. These results may be useful in the future development of breeding programs based on decreasing wool MFD and FDSD, or on increasing wool MSL.

## 1. Introduction

The keratin-associated proteins (KAPs) are structural components of wool and hair fibres. They form a semi-rigid matrix and cross-link with keratin (K) in the keratin intermediate filaments (IFs) [1]. The KAPs are believed to play an important role in defining the physico-mechanical properties of the hair and wool fibres, and variation in the KAPs may therefore underpin variation in wool quality. The KAPs are a complex group of proteins, and they typically possess high levels of cysteine, or glycine and tyrosine. Different KAPs are encoded by different KAP genes, and a large number of KAP genes have been described, clustered on different chromosomes [2]. The KAPs have been allocated into three broad groups according to their amino acid composition: the high sulphur (HS: ≤30 mol% cysteine) KAPs, the ultra-high sulphur (UHS: >0 mol% cysteine) KAPs, and the high glycine–tyrosine (HGT: 35–60 mol% glycine and tyrosine) KAPs [3].

Over one hundred KAP genes (*KRTAPs*) have been identified in different species, and they have been allocated to 27 different gene families [4,5,6]. Of these families, numbers 1–3, 11, 13–16, and 23–27 are HS-KAPs; numbers 4, 5, 9, 10, 12, and 17 are UHS-KAPs; and numbers 6–8 and 18–22 are HGT-KAPs. In humans, there are 12 known HS-KAP families, consisting of 25 functional KAP genes [4,5,6], but only twelve ovine HS-KAP genes from six families have been reported to date [3]. These ovine HS-KAP genes are *KRTAP1-1* to *1-4*, *KRTAP2-1*, *KRTAP2-3*, *KRTAP3-1* to *3-3*, *KRTAP11-1*, *KRTAP13-3*, and *KRTAP24-1*.

The KAP26-1 gene (*KRTAP26-1*) is a HS-KAP. While the gene has been identified in humans [6], it has not been described in any other species.

In this study, we describe the identification of a sequence encoding a putative ovine *KRTAP26-1*, report variation in this gene detected using PCR-single strand conformational polymorphism (PCR-SSCP), and reveal associations between variation in the gene and in some wool traits.

## 2. Materials and Methods

All research involving animals were carried out in accordance with the Animal Welfare Act 1999 (New Zealand Government), and the collection of sheep blood drops by nicking sheep ears is covered by Section 7.5 Animal Identification, of the Animal Welfare (Sheep and Beef Cattle) Code of Welfare 2010; a code of welfare issued under the Animal Welfare Act 1999 (New Zealand Government).

### 2.1. Sheep Blood and Wool Samples

Three hundred and eighty-three Merino × Southdown lambs, the progeny of four different sires, and ninety-four New Zealand (NZ) Romney lambs, sourced from five farms, were used to search for variation in *KRTAP26-1*. The 383 Merino × Southdown cross lambs were subsequently used for the association study. Blood samples from all these sheep were collected onto FTA cards (Whatman BioScience, Middlesex, UK) and genomic DNA was purified using a two-step procedure described by Zhou et al. [7].

Wool samples were collected from the mid-side of the Merino × Southdown-cross lambs at 12 months of age. Greasy fleece weight (GFW) was measured at shearing, and other wool traits were measured by the New Zealand Wool Testing Authority Ltd. (Ahuriri, Napier, NZ). The traits measured included mean fibre diameter (MFD), fibre diameter standard deviation (FDSD), coefficient of variation of fibre diameter (CVFD), mean staple length (MSL), mean fibre curvature (MFC), mean staple strength (MSS), and prickle factor (PF). Wool yield (Yield) was measured, and this was used to calculate the clean fleece weight (CFW).

### 2.2. Search for an Ovine Homologue of Human KRTAP26-1 in the Sheep Genome

A human *KRTAP26-1* sequence (AM941740.1) was used to BLAST search the Ovine Genome Assembly v4.0 (https://blast.ncbi.nlm.nih.gov/). The genome sequences that showed the highest similarity to the human *KRTAP26-1* sequence were presumed to be the ovine *KRTAP26-1*. These sequences were used to design PCR primers for amplifying the entire coding region of this gene from sheep DNA.

### 2.3. PCR Primers and Amplification of Sheep Genomic DNA

The sequences of the PCR primers designed were: 5′-CAGACGACAACCTGCTCCTG-3′ (123641093 to 123641112) and 5′-GTCTGGGATCTTCAGCGTG-3′ (123641700 to 123641718), with the nucleotide positions referring to NC_019458.2. These primers were synthesised by Integrated DNA Technologies (Coralville, IA, USA). PCR amplification was performed in a 15 μL reaction containing the genomic DNA on a 1.2 mm punch of the FTA paper, 0.25 μM primers, 150 μM dNTPs (Bioline, London, UK), 2.5 mM Mg^2+^, 0.5 U of *Taq* DNA polymerase (Qiagen, Hilden, Germany) and the 1× reaction buffer supplied with the enzyme. The thermal profile consisted of 2 min at 94 °C; followed by 35 cycles of 30 s at 94 °C, 30 s at 61 °C, and 30 s at 72 °C; with a final extension of 5 min at 72 °C. Amplification was carried out using S1000 thermal cyclers (Bio-Rad, Hercules, CA, USA).

Amplicons were visualised by electrophoresis in 1% agarose gels (Quantum Scientific, Brisbane, QLD, Australia), using 1× Tris/Borate/EDTA (TBE) buffer (89 mM Tris, 89 mM boric acid, 2 mM Na_2_EDTA) that contained 200 ng/mL of ethidium bromide.

### 2.4. Screening for Variation in KRTAP26-1

PCR amplicons were screened for sequence variation using SSCP analysis. Each amplicon (0.7 μL) was mixed with 7 μL of loading dye (98% formamide, 10 mM EDTA, 0.025% bromophenol blue, 0.025% xylene cyanol). After denaturation at 95 °C for 5 min, the samples were rapidly cooled on wet ice and then loaded on 16 cm × 18 cm, 14% acrylamide/bisacrylamide (37.5:1) (Bio-Rad) gels with 1% glycerol. Electrophoresis was performed using Protean II xi cells (Bio-Rad) in 0.5× TBE buffer, under the following electrophoretic conditions: 32.5 °C at 290 V for 19 h. The gels were silver-stained according to the method of Byun et al. [8].

### 2.5. Sequencing of Allelic Variants and Sequence Analysis

PCR amplicons representing different banding patterns from sheep that appeared to be homozygous were sequenced in both directions three times at the Lincoln University DNA Sequencing Facility, NZ. Variants that were only found in heterozygous sheep were sequenced using an approach described by Gong et al. [9]. Briefly, a band corresponding to the rare variant was excised as a gel slice from the polyacrylamide gel, macerated, and then used as a template for re-amplification with the original primers. This second amplicon was then sequenced three times in both directions. Sequence alignments, translations, and phylogenetic analysis were carried out using DNAMAN (version 5.2.10, Lynnon BioSoft, Vaudreuil, QC, Canada). The phylogenetic tree was constructed using the predicted amino acid sequences for the putative ovine *KRTAP26-1* variants and the human *KRTAP26-1* sequence. All previously identified ovine HS-KAP members were included in the tree, including putative amino acid sequences for *KRTAP1-1*, *KRTAP1-2*, *KRTAP1-3*, *KRTAP1-4*, *KRTAP2-3*, *KRTAP3-1*, *KRTAP3-2*, *KRTAP3-3*, *KRTAP4-1*, *KRTAP4-3*, *KRTAP5-1*, *KRTAP5-4*, *KRTAP5-5*, *KRTAP6-1*, *KRTAP6-2*, *KRTAP6-3*, *KRTAP6-4*, *KRTAP6-5*, *KRTAP7-1*, *KRTAP8-1*, *KRTAP8-2*, *KRTAP11-1*, *KRTAP13-3*, *KRTAP22-1*, and *KRTAP24-1*.

### 2.6. Statistical Analyses

Statistical analyses were performed using Minitab version 16. Firstly, Pearson correlation coefficients were calculated to test the strength of the relationship between the ten wool traits: GFW, CFW, Yield, MFD, FDSD, CVFD, MSL, MSS, CURV, and PF. Next, General Linear Models (GLMs) were used to assess the effect of the presence or absence of the *KRTAP26-1* variants on various wool traits. Initially, single-variant models were performed to ascertain which variants would be included in a second series of multi-variant models, where any variant that had an association with a trait in the single-variant models, with *p* < 0.2, and could thus, potentially impact on the wool trait being tested, were included.

A third series of GLMs were used to compare various wool traits among lambs that had different *KRTAP26-1* genotypes, but only if the genotype frequency was greater than 5%, and thus, represented an adequate sample size. When the GLMs indicated significant differences among genotypes, multiple pairwise comparisons were made with a Bonferroni correction being applied to reduce the chances of obtaining false-positive results during the multiple comparisons.

Sire was found to affect all the wool traits, and hence, was included as a random factor in all models. Gender was found to have an effect on GFW, CFW, MSL, MFD, FSDS, MSS, MFC, and PF, and was therefore included as a fixed factor in the models for these traits. Birth rank had an effect on MSL, and was included as a fixed factor in the model for MSL.

## 3. Results

### 3.1. Correlations between Wool Traits

Correlations were found between many of the wool traits (Table 1).

### 3.2. Identification of KRTAP26-1 in the Sheep Genome

**Figure 1:**
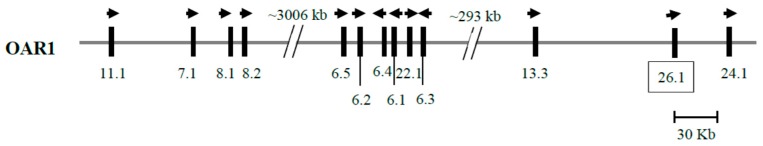
Structure of the sheep chromosome 1 region that contains a sequence similar to human *KRTAP26-1*. The newly identified ovine *KRTAP26-1* is shown in a box, and twelve previously identified *KRTAPs* [3,10] on sheep chromosome 1 are also shown. Vertical bars represent the location of different *KRTAPs* and the arrowheads indicate the direction of transcription. The numbers below the bars indicate the name of the respective keratin-associated protein (KAP) genes (i.e., 11.1 is *KRTAP11-1*). The nucleotide distances refer to NC_019458.

A BLAST search of the Ovine Genome Assembly v4.0 using a human *KRTAP26-1* coding sequence (AM941740.1) revealed a region on sheep chromosome 1 containing a similar sequence to the test sequence. A 555 bp open reading frame was identified between OAR1: 123641123 and 123641677. Three previously identified ovine KAP genes were also found near this open reading frame and from centromere to telomere these were *KRTAP24-1* (123678718 to 123679476), *KRTAP13-3* (123511783 to 123512253), and *KRTAP6-3* (123220969 to 123221184) (Figure 1). Two PCR primers were designed to amplify this gene, and upon sequencing, the amplicons were confirmed to be 626 bp in size.

### 3.3. Variation in Ovine KRTAP26-1

**Figure 2:**
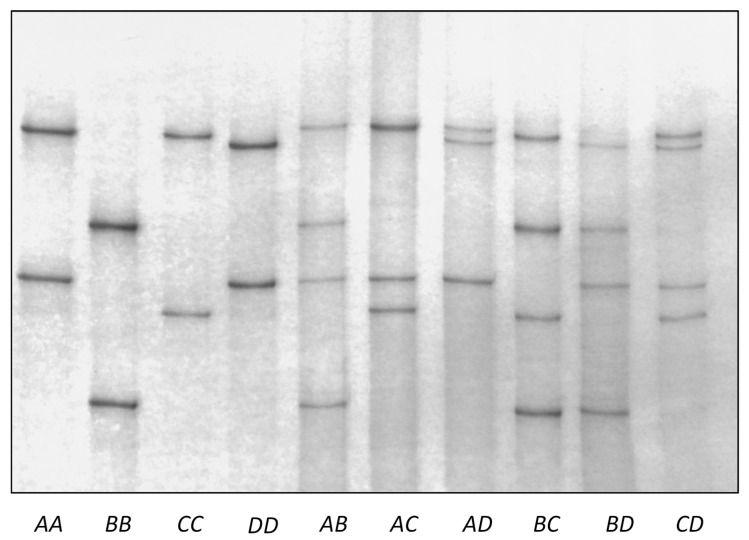
Polymerase chain reaction-single stranded conformational polymorphism (PCR-SSCP) identification of variation in the coding region of ovine *KAP26-1* gene. Four banding patterns, representing four variants (*A* to *D*) are shown, for either homozygous or heterozygous sheep.

**Figure 3:**
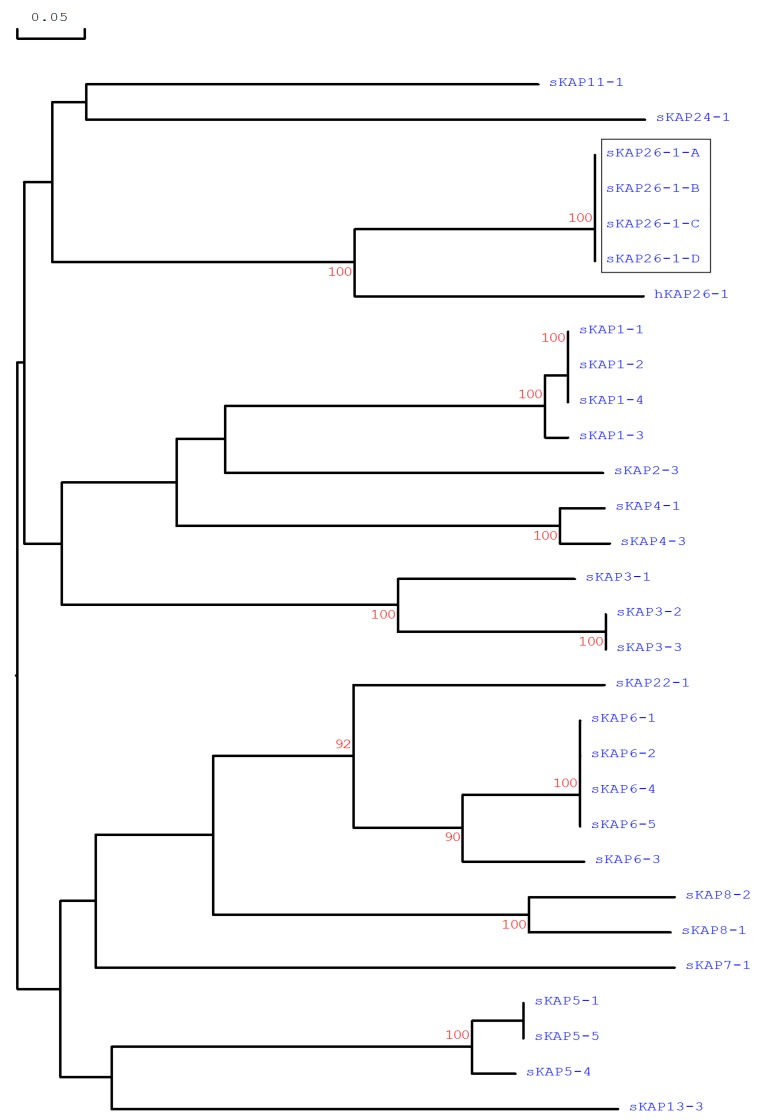
Phylogenetic tree of all the ovine KAPs identified to date together with human KAP26-1. The tree was constructed using the predicted amino acid sequences derived from the genes. The ovine KAPs are indicated with the prefix “s” and the human KAPs have the prefix “h”. The newly identified ovine *KAP26-1* sequences are shown in a box. The numbers at the forks indicate the bootstrap confidence values and only those equal to, or higher than 90%, are shown. The GenBank accession numbers for the human KAP26-1 is AM941740.1. The GenBank accession numbers for the sheep KAPs are X01610, HQ897973, X02925, X01610, U60024, M21099, M21100, M21103, X73462, EU239778, X55294, X73434, X73435, M95719, KT725827, KT725833, KT725838, KT725841, X05638, X05639, KF220646, HQ595347, JN377429, KX377616, and JX112014 for sKAP1-1, sKAP1-2, sKAP1-3, sKAP1-4, sKAP2-3, sKAP3-1, sKAP3-2, sKAP3-3, sKAP4-1, sKAP4-3, sKAP5-1, sKAP5-4, sKAP5-5, sKAP6-1, sKAP6-2, sKAP6-3, sKAP6-4, sKAP6-5, sKAP7-1, sKAP8-1, sKAP8-2, sKAP11-1, sKAP13-3, sKAP22-1, and sKAP24-1, respectively.

There were four PCR-SSCP banding patterns detected for the notional ovine *KRTAP26-1*, with either one or a combination of two banding patterns observed for each sheep (Figure 2). DNA sequencing revealed that these PCR-SSCP patterns represented four nucleotide acid sequences (named *A*, *B*, *C*, and *D*). The sequences were most similar to human *KRTAP26-1* (Figure 3), and were deposited into GenBank with accession numbers KX644903–KX644906. Seven single nucleotide polymorphisms (SNPs) were identified when comparing the four sequences (Table 2). All of these SNPs were located in the coding region, and two of them were non-synonymous substitutions that would result in amino acid changes in the putative KAP26-1 protein.

The ovine *KRTAP26-1* sequences would, if expressed, encode a polypeptide of 184 amino acids. This polypeptide would contain a high content of serine (22.28 mol%), and moderate levels of proline (11.96 mol%), leucine (10.33 mol%), and glycine (8.70 mol%). Other residues that commonly occurred in the polypeptide included cysteine, tyrosine, valine, arginine, and threonine, and these accounted for 8.15, 6.52, 5.43, 4.89, and 4.89 mol%, respectively. The putative KAP26-1 protein would therefore be a basic protein, with a predicted pI value of approximately 7.9.

### 3.4. Comparison of Variant and Genotype Frequencies between NZ Romney and Merino × Southdown-Cross Sheep

The frequencies of the *KRTAP26-1* variants in the NZ Romney sheep were: *A*: 40.4%; *B*: 47.3%; *C*: 0.5%; and *D*: 11.7%; while those in Merino × Southdown-cross sheep were: *A*: 49.6%; *B*: 25.7%; *C*: 23.0%; and *D*: 1.7%. Variant *B* was very common in the NZ Romney sheep, while in Merino × Southdown-cross sheep, *A* was more common. Variant *C* was common in the Merino × Southdown-cross sheep, but was found to be rare in the NZ Romney sheep investigated. The frequencies of *B* and *D* were much higher in the NZ Romney sheep than the Merino × Southdown-cross sheep. Nine genotypes were detected in the Merino × Southdown-cross sheep, and they were as follows: *AA*, *AB*, *AC*, *AD*, *BB*, *BC*, *BD*, *CC*, and *CD*, while *CC* and *CD* were not detected in the NZ Romney sheep. Genotype *DD* was found in the NZ Romney sheep. Of the genotypes present in the Merino × Southdown-cross sheep, only five (*AA*, *AB*, *AC*, *BB* and *BC*) occurred at a frequency over 5%, and these were used in the genotype association analyses.

### 3.5. Effect of Variation in KRTAP26-1 on Wool Traits

Of the four *KRTAP26-1* variants detected in Merino × Southdown-cross lambs, *D* occurred at a very low frequency, and so its association with wool traits was not tested.

In the single-variant models, the presence of *B* was associated with an increase in MFD, MFC, and PF (Table 3). The effect of *B* on these traits was lost when *C* was introduced into models, but trends were still evident for MFD (*p* = 0.081) and MFC (*p* = 0.056). The presence of *C* was found to be associated with an increase in Yield and MSL; and a decrease in MFD, FDSD, and PF in the single-variant models. These effects on MFD, FDSD, and PF persisted, when corrected for *B*, in the multi-variant models.

### 3.6. Effect of Common Genotypes on Wool Traits

With the five common *KRTAP26-1* genotypes (*AA*, *AB*, *AC*, *BB*, and *BC*), an effect of genotype was observed for MFD, FDSD, MSL, and PF (Table 4). Sheep of genotypes *AB* and *BB* were of a predicted mean MFD, from 5.8% to 11.2% higher than those of genotypes *AC* and *BC*. Genotypes *AA*, *AB*, and *BB* were associated with a higher predicted mean FDSD, than *AC* and *BC*. Genotypes *AC* and *BC* were associated with a higher predicted mean MSL over 6% greater than *AB* and *BB*. In terms of PF, *AB* and *BB* were associated with a higher predicted mean PF compared to *AC* and *BC*; especially sheep with *BB*, which had a predicated mean PF of 5.04%, over 2.7 times higher than that of sheep with *AC* and *BC*.

## 4. Discussion

The phenotypic correlations observed among the various wool traits were similar to other studies [11,12,13], but with some exceptions. The phenotypic correlations between CVFD and GFW, CFW, or MSL were −0.304, −0.334, and −0.306, respectively. These are higher than correlations described by Gong et al. [11], and Huisman and Brown [13]. The phenotypic correlation between CVFD and MSS was −0.290, while Huisman and Brown [13] reported a figure of −0.66. These differences may be a consequence of the different ages, genders, and breeds of sheep being studied. For example, Huisman and Brown [13] reported correlations between MSL and MFC in yearling, hogget and adult Merino sheep of −0.38, −0.48, and −0.52, respectively, while in this study it was −0.442 in yearling Merino-cross sheep. FDSD, which provides an indication of wool quality, was strongly correlated with MFD, CVFD, and PF in this study, which is perhaps unsurprising.

The putative ovine *KRTAP26-1* was located on OAR1. The gene was clustered with several previously described KAP genes, and displayed a lower sequence similarity to any previously described ovine KAP gene, when compared to a *KRTAP26-1* sequence from humans. The identification of this ovine *KRTAP26-1* sequence lifts the number of HS-KAP members identified in sheep from 12 to 13.

The peptide that would be produced from the ovine *KRTAP26-1* sequence would contain 184 amino acids. Although human KAP26-1 has been classed as a HS-KAP [6], its cysteine content (8.15%) would be lower than any other high-sulphur ovine KAP protein. This does not sit that comfortably with the current KAP classification system, as the protein would be neither cysteine-rich, nor glycine- and tyrosine-rich. The putative ovine KAP26-1 protein would contain a very high content of serine (22.28%), which reflects what has been reported for other HS-KAPs, such as KAP24-1 [14], KAP11-1 [15], and KAP13-3 [16]. There would be a serine-rich region (amino acids 121 to 124) in KAP26-1, which has not been described in any other ovine KAP proteins described to date. Serine residues can be phosphorylated, so having large amounts of serine may be associated with the phosphorylation of this protein. While phosphorylation has not been detected on wool KAPs, in hair keratins and matrix proteins, phosphorylation and dephosphorylation may affect the organisation and structure of the microfibers [17].

With the PCR-SSCP method, sequence variations were detected in a 626 bp amplicon. It is thought that PCR-SSCP analysis is better suited to small fragments in the range of 150–200 base pairs, but it has been shown to detect variation in larger fragments (up to 640 base pairs) under optimised conditions [18,19,20]. The addition of glycerol to SSCP gels can improve the sensitivity of analysis, and allow an increase in the length of DNA fragments that can be analysed [21]. Our results with 1% glycerol in the gels would appear to support this contention.

Using the PCR-SSCP method, seven SNPs were detected in the ovine *KRTAP26-1* sequences and these produced four unique sequence variants. Among the seven SNPs, two of the substitutions would notionally result in amino acid changes. One of them was detected in *B*: c.215G/A (p.72C/Y), while the other was in *C*: c.277A/G (p.93S/G). The variation found in *B*, when compared to the other variants, would result in loss or gain of cysteine, which may affect the protein’s ability to cross-link with the intermediate filaments, or other KAPs. The variation found in *C*, when compared to the other variants, would result in the loss or gain of serine and glycine, which may also potentially affect the phosphorylation of this KAP.

Sequence variation in *KRTAP26-1* was found to be associated with a number of wool traits, including Yield, MFD, FDSD, MSL, MFC, and PF. However, the largest and most enduring effect appeared to be on the fibre diameter associated traits of MFD, FDSD, and PF. Large differences in the predicated means were observed between the common genotypes, and differences that reinforced the conclusions drawn from the variant absence/presence models.

The results suggest that sheep with *C* have on average higher wool quality, as they have lower predicted mean MFD, FDSD and PF, and higher Yield and MSL. This is consistent with the correlations found between these traits. The correlations between MFD, FDSD, and PF were strong and positive, while these three traits had moderate negative correlations with Yield and MSL. Wool of lower MFD, and reduced FDSD in combination with higher MSL, would be described as being of higher quality and be more desirable in the market [22].

The presence of *B* seems to have an opposite effect to *C*, with these sheep having higher predicted mean MFD and PF. The wool traits of sheep with *A* were between those that have either *B* or *C*. The association analysis results were also consistent with the gene frequencies observed in the two sheep breeds. Of the four variants observed; *B*, *C*, and *D* had large differences in frequency between the two breeds. Merino × Southdown-cross sheep (MFD below 21 µm, Table 3) characteristically have lower MFD than Romney sheep (MFD of 33–37 µm) [23]. This may in part explain the difference in the frequency of the variants, between Merino × Southdown-cross sheep and Romney sheep, although the possibility exists that the effects observed for *KRTAP26-1* may be due to its linkage to the other *KRTAPs* in the location of the gene and/or other genes.

## Figures and Tables

**Table 1:** Pearson correlation coefficients between various wool traits (*n* = 384 Merino × Southdown-cross sheep).

	GFW	CFW	Yield	MSL	MFD	FDSD	CVFD	MSS	MFC
CFW	**0.916** ***								
Yield	0.165 **	0.528 ***							
MSL	0.463 ***	0.525 ***	0.341 ***						
MFD	0.053	−0.091	−0.341 ***	−0.270 ***					
FDSD	0.160 **	0.263 ***	0.309 ***	0.350 ***	**0.796** ***				
CVFD	0.304 ***	0.334 ***	0.174 **	0.306 ***	0.306 ***	**0.815** ***			
MSS	0.188 ***	0.220 ***	0.143 ***	0.067	0.069	−0.143 **	0.290 ***		
MFC	0.329 ***	0.492 ***	0.550 ***	0.442 ***	0.425 ***	0.397 ***	0.238 ***	0.079	
PF	0.022	0.085	0.251 ***	0.244 ***	0.819 ***	0.794 ***	0.440 ***	0.061	0.224 ***

Correlations with |*r*| > 0.7 are in bold, and those with 0.3 <|*r*| ≤ 0.7 are underlined. ** *p* < 0.01. *** *p* < 0.001. GFW: Greasy fleece weight; CFW: clean fleece weight; Yield: wool yield; MSL: mean staple length; MFD: mean fibre diameter; FDSD: fibre diameter standard deviation; CVFD: coefficient of variation of fibre diameter; MSS: mean staple strength; MFC: mean fibre curvature.

**Table 2:** Sequence variations identified in the four variants of ovine *KRTAP26-1*.

Nucleotide Position	Variant				Amino Acid Change
	KRTAP26-1*A	KRTAP26-1*B	KRTAP26-1*C	KRTAP26-1*D	
c.72	C	T	C	C	No change
c.111	A	C	T	A	No change
c.123	C	G	C	C	No change
c.215	G	A	G	G	p.Cys72Tyr
c.277	A	A	G	A	p.Ser93Gly
c.336	G	G	G	A	No change
c.549	C	T	C	C	No change

**Table 3:** Association between the *KRTAP26-1* variants and various wool traits (predicted mean ± predicted SE) ^1^.

Trait	Variant Assessed	*n*		Single-Variant Model			Multi-Variant Model			
		Absent	Present	Absent	Present	*p*	Variants Fitted	Absent	Present	*p*
GFW	*A*	71	312	2.37 ± 0.06	2.37 ± 0.03	0.958				
(kg)	*B*	207	176	2.37 ± 0.04	2.37 ± 0.04	0.902				
	*C*	212	171	2.39 ± 0.03	2.33 ± 0.05	0.361				
CFW	*A*	71	312	1.72 ± 0.05	1.71 ± 0.02	0.764				
(kg)	*B*	207	176	1.72 ± 0.03	1.70 ± 0.03	0.678				
	*C*	212	171	1.71 ± 0.03	1.72 ± 0.04	0.877				
Yield	*A*	71	312	72.2 ± 0.74	71.7 ± 0.35	0.471	*B*, *C*	73.0 ± 0.82	71.8 ± 0.41	0.201
(%)	*B*	207	176	*72.4 ± 0.47*	*71.2 ± 0.44*	*0.070*	*C*	72.4 ± 0.47	71.7 ± 0.52	0.291
	*C*	212	171	**71.2 ± 0.40**	**73.0 ± 0.65**	**0.027**	*B*	*71.3 ± 0.41*	*72.8 ± 0.68*	*0.082*
MSL (mm)	*A*	71	312	84.7 ± 1.66	82.6 ± 0.87	0.253	*C*	85.6 ± 1.64	84.0 ± 0.93	0.377
	*B*	207	176	83.9 ± 1.12	82.2 ± 1.04	0.236	*C*	84.3 ± 1.10	84.4 ± 1.18	0.950
	*C*	212	171	**80.7 ± 0.99**	**87.9 ± 1.51**	**<0.001**	None	**80.7 ± 0.99**	**87.9 ± 1.51**	**<0.001**
MFD	*A*	71	312	19.8 ± 0.25	19.6 ± 0.13	0.375	*B*, *C*	19.6 ± 0.27	19.3 ± 0.14	0.397
(µm)	*B*	207	176	**19.3 ± 0.17**	**19.9 ± 0.15**	**0.001**	*C*	*19.2 ± 0.16*	*19.6 ± 0.17*	*0.081*
	*C*	212	171	**20.1 ± 0.15**	**18.6 ± 0.22**	**<0.001**	*B*	**20.0 ± 0.15**	**18.7 ± 0.23**	**<0.001**
FDSD	*A*	71	312	4.26 ± 0.09	4.22 ± 0.04	0.680	*B*, *C*	4.21 ± 0.09	4.13 ± 0.04	0.450
(µm)	*B*	207	176	*4.16 ± 0.06*	*4.28 ± 0.05*	*0.098*	*C*	4.13 ± 0.06	4.17 ± 0.06	0.626
	*C*	212	171	**4.35 ± 0.05**	**3.96 ± 0.08**	**<0.001**	*B*	**4.34 ± 0.05**	**3.96 ± 0.08**	**<0.001**
CVFD	*A*	71	312	21.4 ± 0.28	21.9 ± 0.14	*0.166*	*C*	21.4 ± 0.28	21.7 ± 0.15	0.248
(%)	*B*	207	176	21.9 ± 0.18	21.7 ± 0.17	0.471	*A*, *C*	21.7 ± 0.26	21.4 ± 0.20	0.330
	*C*	212	171	*22.0 ± 0.16*	*21.4 ± 0.25*	*0.067*	*A*	*21.8 ± 0.19*	*21.3 ± 0.26*	*0.093*
MSS	*A*	71	312	23.9 ± 1.06	22.6 ± 0.55	0.237	*B*	23.6 ± 1.19	22.7 ± 0.55	0.496
(N/ktex)	*B*	207	176	22.1 ± 0.71	23.4 ± 0.65	*0.145*	None	22.1 ± 0.71	23.4 ± 0.65	*0.145*
	*C*	212	171	23.1 ± 0.64	22.2 ± 0.98	0.434	*B*	22.9 ± 0.66	22.6 ± 1.01	0.774
MFC	*A*	71	312	91.2 ± 1.80	89.5 ± 0.85	0.367	*B*, *C*	89.4 ± 2.00	89.1 ± 1.01	0.862
(°/mm)	*B*	207	176	**87.7 ± 1.15**	**91.4 ± 1.06**	**0.016**	*C*	*87.6 ± 1.16*	*90.7 ± 1.26*	*0.056*
	*C*	212	171	90.8 ± 0.98	87.2 ± 1.60	*0.070*	*B*	90.3 ± 1.02	88.0 ± 1.65	0.278
PF	*A*	71	312	3.00 ± 0.45	2.79 ± 0.23	0.654	*B*, *C*	2.55 ± 0.49	2.44 ± 0.26	0.845
(%)	*B*	207	176	**2.33 ± 0.30**	**3.24 ± 0.27**	**0.017**	*C*	2.23 ± 0.29	2.69 ± 0.31	0.237
	*C*	212	171	**3.47 ± 0.26**	**1.41 ± 0.40**	**<0.001**	*B*	**3.39 ± 0.27**	**1.53 ± 0.41**	**<0.001**

^1^ Predicted means and predicted standard errors derived from GLMs with various factors being included in the models, as described in the Materials and Methods. *p* < 0.05 are in bold, while 0.05 ≤ *p* < 0.20 are italicised.

**Table 4:** The effect of *KRTAP26-1* genotype on various wool traits (predicted mean ± predicted SE) ^1^.

Trait	AA (*n* = 68)	AB (*n* = 116)	AC (*n* = 123)	BB (*n* = 22)	BC (*n* = 37)	*p*
GFW (kg)	2.39 ± 0.05	2.36 ± 0.04	2.36 ± 0.05	2.39 ± 0.09	2.40 ± 0.08	0.971
CFW (kg)	1.72 ± 0.04	1.68 ± 0.03	1.72 ± 0.04	1.69 ± 0.07	1.77 ± 0.06	0.814
Yield (%)	73.5 ± 1.32	72.7 ± 1.22	74.6 ± 1.28	72.7 ± 1.65	74.5 ± 1.58	0.310
MSL (mm)	82.6 ± 1.41 ^a,b^	81.2 ± 1.07 ^b^	86.5 ± 1.45 ^a^	78.2 ± 2.48 ^b^	88.2 ± 2.17 ^a^	**0.002**
MFD (µm)	19.7 ± 0.22 ^a,b^	20.2 ± 0.17 ^a^	18.7 ± 0.23 ^c^	20.8 ± 0.39 ^a^	19.1 ± 0.34 ^b,c^	**<0.001**
FDSD (µm)	4.41 ± 0.08 ^a^	4.46 ± 0.06 ^a^	4.14 ± 0.08 ^b^	4.67 ± 0.13 ^a^	4.15 ± 0.12 ^b^	**0.001**
CVFD (%)	22.3 ± 0.26	22.0 ± 0.20	22.0 ± 0.27	22.4 ± 0.47	21.7 ± 0.41	0.624
MSS (N/ktex)	22.1 ± 0.89	22.7 ± 0.68	21.4 ± 0.92	25.6 ± 1.58	22.7 ± 1.38	0.202
MFC (o/mm)	89.1 ± 1.76	91.8 ± 1.31	87.0 ± 1.69	92.1 ± 3.01	88.8 ± 2.61	0.260
PF (%)	3.21 ± 0.40 ^a^^,^^b^	3.50 ± 0.32 ^a^	1.36 ± 0.15 ^b^	5.04 ± 1.08 ^a^	1.39 ± 0.24 ^b^	**<0.001**

^1^ Predicted means and predicted standard errors derived from GLMs with various factors being included in the models as described in the Materials and Methods. Means within rows that do not share a superscript letter were different at *p* < 0.05 and the *p*-values bolded.
